# Porphyria Cutanea Tarda Associated With Acute Hemorrhagic Pancreatitis

**DOI:** 10.1177/2324709619852769

**Published:** 2019-06-03

**Authors:** Manasi Singh, Ashley Duckett, Marc Heincelman

**Affiliations:** 1 Medical University of South Carolina, Charleston, SC, USA

**Keywords:** porphyria cutanea tarda, acute hemorrhagic pancreatitis

## Abstract

Porphyria cutanea tarda (PCT) is a condition of dysregulated heme synthesis that leads to accumulation of photosensitizing precursors with resultant fragility and blistering of the skin. It can be hereditary or acquired and has been known to be associated with hepatic C virus, alcohol, HIV, and estrogen. In this article, we report an unusual presentation of PCT associated with acute hemorrhagic pancreatitis in a 57-year-old man. He presented initially to a community hospital with acute onset of epigastric abdominal pain and new-onset ascites. Lipase was elevated. Diagnostic paracentesis was grossly bloody. He was then transferred to our institution for concern for acute hemorrhagic pancreatitis. On arrival, physical examination demonstrated vesicles and bullae with erythematous bases, in different stages of healing seen over the dorsal aspects of both hands with scaling, scarring, and hypopigmentation and hyperpigmentation of the skin. Laboratory evaluation and skin biopsy confirmed the diagnosis of PCT. Search for an underlying etiology failed to reveal typical predisposing factors. This report illustrates that acute hemorrhagic pancreatitis may be an underlying etiology for PCT.

## Case Presentation

A 57-year-old previously healthy Caucasian male presented to a community hospital with a 3-day onset of epigastric abdominal pain, nausea, vomiting, and new-onset ascites. Initial workup revealed normal liver function studies. His lipase was elevated. Diagnostic paracentesis was consistent with hemorrhagic fluid with red blood cell count of 640 504/mm^3^ and white blood cell count of 1440/mm^3^. Computed tomography scan of the abdomen was concerning for a 15 mm lesion in the pancreatic head, peripancreatic stranding, and large-volume ascites. He was transferred to this tertiary care hospital for further workup of acute hemorrhagic pancreatitis and possible endoscopic ultrasound/endoscopic retrograde cholangiopancreatography for further workup of the pancreatic head lesion.

He had been in good health prior to this presentation. Past medical history was pertinent for hypertension and chronic obstructive pulmonary disease. He had no history of pancreatitis, gallstones, or other hepatobiliary disease. Social history revealed that he was active, independent, and lived by himself. He had 1 rum drink daily and smoked 2 cigarettes per day. Review of systems was pertinent for blistering of the hands, with subsequent scabbing and pigmentation changes, with onset a few days prior to the abdominal pain. He was not sure if there was a relationship to sun exposure. Associated with the blisters, the patient described unrelenting pruritis of his hands bilaterally. This was the first time in his life he was experiencing cutaneous symptoms as he had never had skin problems before.

**Figure 1.**
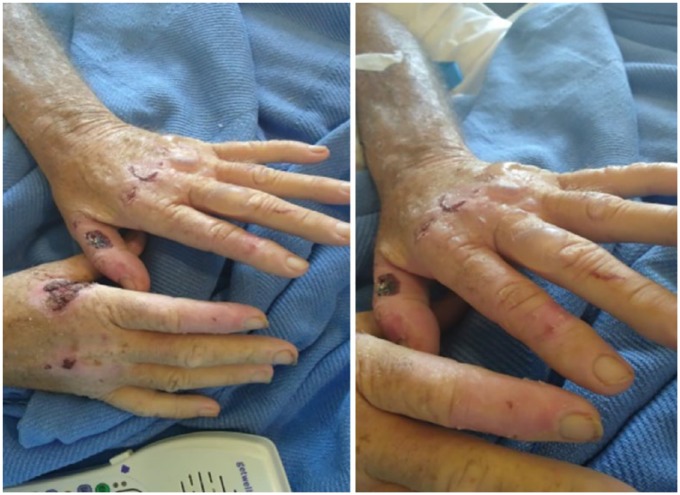
Skin findings. Tense bullae and hemorrhagic crusting erosions with scarring on dorsum of both hands.

On admission to our institution, vital signs demonstrated temperature 37.0°C, blood pressure 146/95 mm Hg, heart rate 112 beats per minute, respiratory rate 20 breaths per minute, and oxygen saturation 96% on room air. On examination, he was in mild distress from abdominal pain and nausea. Abdomen was distended and mildly tender in the epigastric region. A fluid wave was appreciated. There was no hepatosplenomegaly. Dermatology examination revealed vesicles and bullae with erythematous bases, in different stages of healing seen over the dorsal aspects of the both hands with scaling, scarring, and hypopigmentation and hyperpigmentation of the skin (Figure 1). They were pruritic but not painful. There was no hypertrichosis appreciated. There were no stigmata of chronic liver disease and no scleral icterus.

Complete blood count demonstrated hemoglobin of 8.5 gm/dL (mean corpuscular volume 95 fL), white blood cell count of 10 500/mm^3^ (76% polymorphonuclear cells), and platelets 328 000/mm^3^. Renal function tests were normal. Liver function tests demonstrated total bilirubin 0.3 mg/dL, aspartate aminotransferase 16 U/L, alanine aminotransferase 7 U/L, alkaline phosphatase 88 U/L, and albumin 1.6 g/dL. The international normalized ratio was 1.35. Lipase was elevated to 352 U/L. Repeat diagnostic paracentesis again demonstrated grossly bloody fluid with an amylase of 2866 U/L.

Further workup for acute hemorrhagic pancreatitis included a computed tomography angiogram, which did not show active bleeding. The nature of the pancreatic head lesion was still indeterminate so an endoscopic ultrasound was performed, which did not show any focal lesion concerning for malignancy. Endoscopic retrograde cholangiopancreatography done showed a large proximal pancreatic duct disruption, which was treated with a plastic stent. He was managed supportively with intravenous fluids and pain relief for acute hemorrhagic pancreatitis.

**Figure 2.**
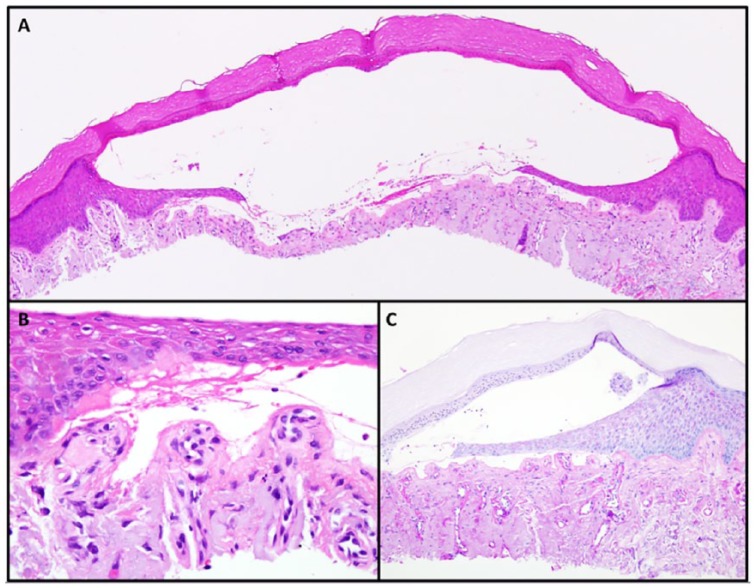
Pathology findings. Hematoxylin and eosin (H&E) demonstrating a cell-poor subepidermal blister (4×). (B) H&E demonstrating festooning (40×). (C) Diastase periodic acid–Schiff (PAS) stain highlighting perivascular PAS positive deposition within superficial dermis (10×).

Further workup of his skin lesions was initiated due to the concern for porphyria cutanea tarda (PCT). Skin biopsy showed blistering dermatosis, subepithelial with evidence of re-epithelialization. Periodic acid–Schiff stain (PAS) with diastase highlighted increased PAS+ deposition around vessel walls and focal PAS+ deposits within the epidermis (Figure 2). Direct immunofluorescence demonstrated homogenously thickened dermal blood vessels highlighted by immunoglobulin (Ig)G, IgA, C1q, fibrin, kappa, and lambda. The specimen was negative for deposition of IgM and C3. Findings were consistent with PCT. Serum porphyrin levels came back strongly positive with levels of 8 µg% (upper limit of normal <0.9 µg%). Fractionation revealed high heptacarboxyl, hexacarboxyl, and pentacarboxyl porphyrins as expected with the heptacarboxyl highest at 2.6 µg% and the other 2 at 1.4 µg% each. After confirmation of the diagnosis, hepatitis C, hepatitis B, and HIV were ordered and returned negative.

Unfortunately, our patient developed secondary bacterial peritonitis and quickly deteriorated. His repeat ascitic fluid grew *Candida* and *Enterococcus*. His respiratory status declined as he developed acute respiratory distress syndrome and needed mechanical ventilation. He then progressed to multiorgan failure a few days later and eventually died.

## Discussion

**Figure 3.**
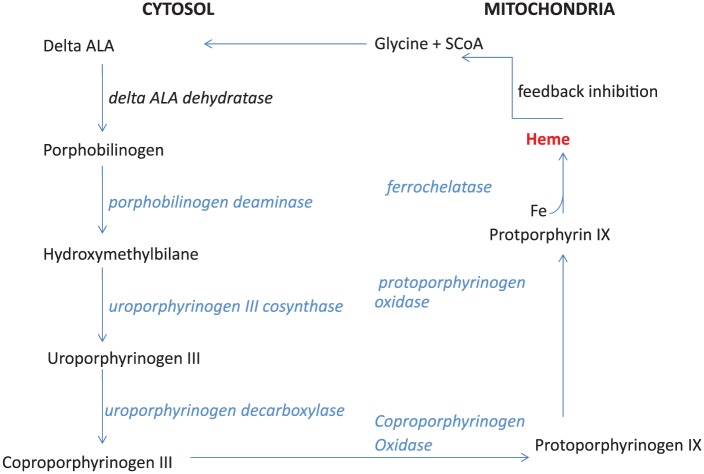
Heme synthesis pathway.

Our patient was diagnosed with PCT associated with acute hemorrhagic pancreatitis. In general, porphyrias are metabolic disorders that affect the heme biosynthesis pathway. Heme biosynthesis involves 8 enzymatic steps in the conversion of glycine and succinyl-CoA to heme (Figure 3). A deficiency of any specific enzyme leads to precursor build up at the previous step with its excretion into bile or urine. Porphyrias are classified as hepatic or photocutaneous porphyrias, depending on the main site of overproduction and accumulation of porphyrins.^1^ PCT occurs from deposition of photosensitizing porphyrins in the skin when the UROD enzyme in the heme synthetic pathway is deficient. Skin lesions, dyspigmentation, hypertrichosis, and dark urine can be seen. Clinical manifestation occurs in the fourth or fifth decades of life, sometimes earlier. Incidence of PCT varies within countries ranging from 1:5000 to 1:70 000.^2,3^ Our patient was 57 –years old at the time of diagnosis.

The pathogenesis of PCT is complex but the final common pathway involves hepatic iron loading and increased oxidative stress.^4^ Almost 80% of cases of PCT are sporadic rather than hereditary with iron overload states, heavy alcohol use, hepatic C virus (HCV), HIV, and exposure to polychlorinated hydrocarbons being common associations due to acquired enzyme inhibition. Iron deficiency seems to be protective in both humans and animal models. Nearly half the patients with PCT carry the HFE gene, and serial phlebotomy is the treatment with removal of blood every 2 weeks for a few months until ferritin is less than the lower limit of normal range.^5^ A study of hemochromatosis genes and other factors contributing to PCT done by Bulaj et al showed that excessive alcohol consumption was more common in men than women with PCT and in those with coinfections with HCV or HIV.^6-8^ Another study of risk factors done by Munos-Santos in Spain of 152 patients also showed a high prevalence of hepatitis C virus infection (65.8%) and alcohol abuse (59.9%), both more frequently in men.^9^ A study of 1613 non-porphyric adults showed a significant positive association of alcohol intake and porphyrinuria.^10^ Interestingly, our patient used alcohol and tobacco but neither in excess. Furthermore, he was relatively iron deficient and HCV and HIV were negative.

Almost all patients have 3 predisposing factors, which bring the UROD levels down to 80% of normal, the level needed for clinical features to manifest.^7,11-14^ These risk factors also contribute by reducing hepcidin production by the hepatocytes causing more iron absorption. Deficiency of the enzyme results in accumulation of excess porphyrins, which are excited by light at 410 nm and injure the skin on returning to their baseline energy state.^10^ The injury can thus be delayed and the association between sunlight and the blistering may be missed by patients, including the patient described in this case.

The diagnosis of PCT can be confirmed by high serum porphyrins, increased excretion of urinary uroporphyrin, and fecal coproporphyrin and isocoproporphyrin.^15^ Normally, only trace amounts of porphyrins are present in plasma, and the amounts increase markedly in patients with cutaneous porphyrias. Being both sensitive and specific, it is increased in any patient with skin problems related to any type of porphyria and is seldom increased in other conditions. If serum porphyrins are negative, porphyrias as a cause of the blistering condition can be ruled out.^15^

In summary, our patient presented with blistering skin lesions in the setting of acute hemorrhagic pancreatitis with elevated porphyrin levels and a biopsy congruent with PCT. Of note, he had no known predisposing risk factors associated with PCT. This leads us to question if acute pancreatitis can act as an oxidative stressor to tip the enzymatic balance and make PCT manifest. To our knowledge, this is the first reported case of PCT in a patient of acute hemorrhagic pancreatitis. It would be interesting to observe if more such associations are seen implicating hemorrhagic pancreatitis as the oxidative stressor leading to the manifestation of PCT.
